# Targeted Disruption of ALK Reveals a Potential Role in Hypogonadotropic Hypogonadism

**DOI:** 10.1371/journal.pone.0123542

**Published:** 2015-05-08

**Authors:** Barbara Witek, Abeer El Wakil, Christoffer Nord, Ulf Ahlgren, Maria Eriksson, Emma Vernersson-Lindahl, Åslaug Helland, Oleg A. Alexeyev, Bengt Hallberg, Ruth H. Palmer

**Affiliations:** 1 Department of Medical Biochemistry and Cell Biology, Institute of Biomedicine, Sahlgrenska Academy, University of Gothenburg, Gothenburg, Sweden; 2 Department of Molecular Biology, Umeå University, Umeå, Sweden; 3 Umeå Center for Molecular Medicine, Umeå University, Umeå, Sweden; 4 Institution for Medical Biosciences/Pathology, Umeå University, Umeå, Sweden; 5 Department of Oncology, Oslo University Hospital Radiumhospitalet, Oslo, Norway; Clermont Université, FRANCE

## Abstract

Mice lacking ALK activity have previously been reported to exhibit subtle behavioral phenotypes. In this study of ALK of loss of function mice we present data supporting a role for ALK in hypogonadotropic hypogonadism in male mice. We observed lower level of serum testosterone at P40 in ALK knock-out males, accompanied by mild disorganization of seminiferous tubules exhibiting decreased numbers of GATA4 expressing cells. These observations highlight a role for ALK in testis function and are further supported by experiments in which chemical inhibition of ALK activity with the ALK TKI crizotinib was employed. Oral administration of crizotinib resulted in a decrease of serum testosterone levels in adult wild type male mice, which reverted to normal levels after cessation of treatment. Analysis of GnRH expression in neurons of the hypothalamus revealed a significant decrease in the number of GnRH positive neurons in ALK knock-out mice at P40 when compared with control littermates. Thus, ALK appears to be involved in hypogonadotropic hypogonadism by regulating the timing of pubertal onset and testis function at the upper levels of the hypothalamic-pituitary gonadal axis.

## Introduction

The Anaplastic Lymphoma Kinase (ALK) Receptor Tyrosine Kinase (RTK) was originally discovered as a fusion protein together with Nucleophosmin (NPM) in anaplastic large-cell non-Hodgkin’s lymphoma (ACLC) in 1994 [1]. The full length receptor was later cloned revealing a transmembrane receptor tyrosine kinase, most similar to those of the Insulin Receptor family [2, 3]. The oncogenic properties of NPM-ALK are thought to arise from the ability of NPM to dimerize thereby mediating constant activation of the ALK kinase domain [4, 5]. Since discovery of the fusion NPM-ALK protein, more than 20 ALK fusion partners have been observed not only in ALCL but also in diseases such as inflammatory myofibroblastic tumor (IMT), non-small cell lung cancer (NSCLC), renal carcinoma, breast cancer, colon carcinoma, serous ovarian carcinoma, oesophageal squamous cell carcinoma (ESCC) and diffuse large B cell lymphoma (DLBLC) [4]. In addition to the numerous translocation events, gain of function ALK mutations have been observed in both spontaneous and hereditary neuroblastoma [4, 6–10].

The expression pattern of ALK in vertebrates has been described in several studies, where ALK has been shown to be expressed in the CNS and the PNS, as well as in testis and ovary [2, 3, 11]. Despite this, deletion of *ALK* in mice does not result in serious phenotypes and the physiological role of ALK in mammals is unclear [12, 13]. On closer examination however, mild behavioral phenotypes have been observed, such as increased struggle time (as measured with tail suspension and Porsott swim tests), enhanced performance in novel object-recognition test and enhanced spatial memory [12, 13]. Recent reports have described interesting side effects in patients treated with the FDA approved ALK inhibitor crizotinib [14], which include reduced hearing, suppression of testosterone levels in men and visual disturbances [15–17]. While these side effects are reversible upon withdrawal of therapeutic treatment of patients it is unclear how much is specific to inhibition of ALK activity.

Puberty is defined as a physiological and developmental process towards sexual maturity. Onset of puberty is initiated by neuroendocrine events that activate the pulsatile release of GnRH from the hippocampus into the hypophyseal portal blood system to stimulate the synthesis and secretion of gonadotropins from anterior pituitary cells. Gonadotropins, in turn, bind to ligand-specific receptors in the gonads, causing gonadal maturation and production of sex steroids, most notably testosterone in males [18–20]. Hippocampal GnRH neurons originate in the nasal placode and migrate through the nasal compartment and the cribriform plate and finally pass through the basal forebrain, before reaching the hypothalamus [19, 21, 22]. Output from these neurons is critical for initiation of puberty as well as maintenance of fertility. A critical role for GnRH neuronal activity in puberty is highlighted by infertility in mice with defective GnRH biosynthesis [23].

Herein we describe the investigation of an ALK knock-out mouse model in which the kinase domain encoding exons have been removed. In agreement with previous studies [12, 13], we confirm that homozygous ALK mutant animals are viable and fertile and do not exhibit any gross morphological defects either during embryogenesis or as adult animals. Targeted disruption of ALK results in decreased levels of serum testosterone at 40 days of age when compared with controls. Further examination reveals that ALK mutant male mice display mild changes in testicular tissue organization at P40. In addition, male ALK KO mice display a delay in pubertal onset as measured by preputial separation. Moreover, the number of gonadotropin releasing hormone positive neurons in the hippocampus of ALK mutant mice is significantly reduced when compared with controls. Furthermore, we are able to recapitulate in wild type mice the effects of crizotinib treatment in lung cancer patients, as inhibition of ALK activity by crizotinib treatment results in a reduction of testosterone levels in adult wild type male mice, as has been observed in crizotinib treated male patients. Taken together, the data presented here suggests that ALK plays a potential role in hypogonadotropic hypogonadism in males.

## Material and Methods

### Ethical permission

All *in vivo* experiments were conducted with approved Swedish legal ethical committee permits A203-12 and A155-12.

### Targeting vector construction and generation of ALK Kinase knock-out mice

**Fig 1:**
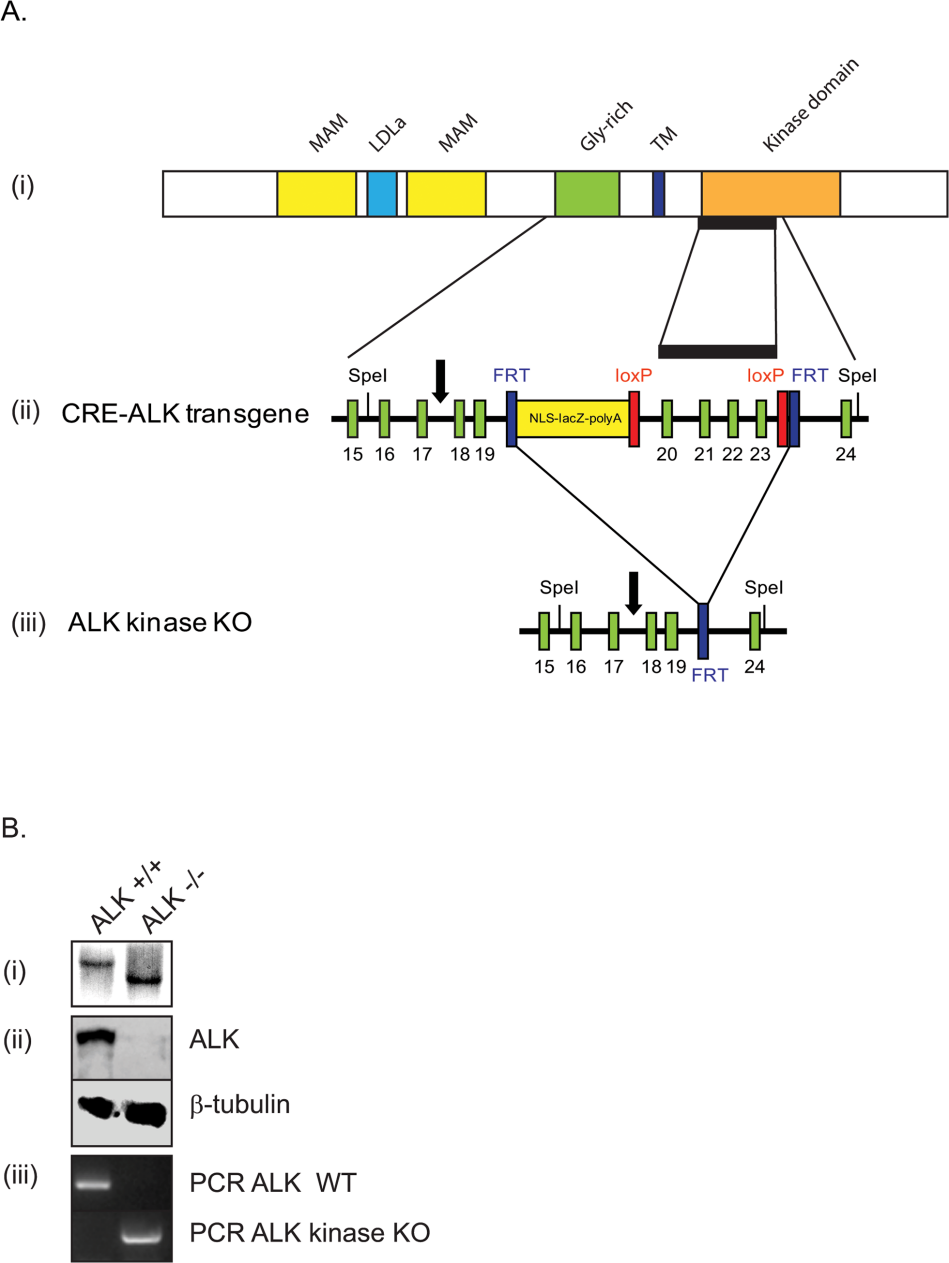
Generation and verification of the ALK kinase knock-out mouse strain. (A) Schematic overview of the ALK kinase knock-out mice. (i) Schematic representation of ALK protein structure. The region of the protein corresponding to the targeted exons 20–23 is indicated (black bar). (ii) Schematic overview of the CRE-ALK construct (conditional knock-out). FRT (blue) and loxP (red) sites are indicated. Restriction sites for Southern are marked with the restriction enzyme SpeI, black arrows indicate the position of the Southern probe used for genotyping. Green bars denoted with the numbers 15–22 respectively are exons. (iii) Schematic overview of ALK kinase KO (non-conditional knock-out). Flp-mediated recombination leads to the deletion of exons 20–23. The remaining FRT site still present after Flp mediated recombination. (B) Verification of the ALK kinase KO line by (i) southern blot, (ii) immunobloting with anti-ALK mAb135 which recognizes the extracellular domain of ALK and by (iii) PCR.

PolyGene Transgenetic (Polygene AG, Riedmattstrasse 9, CH-Rümlang, Switzerland, www.polygene.ch) generated the ALK kinase KO according to our specifications. A gene targeting vector for ALK was designed to generate a floxed allele of ALK with the goal of eliminating exon 20–23. Flipase Recognition Target (FRT)-sites were included in the vector to generate a non-conditional knock-out lacking exon 20–23 (kinase domain). This non-conditional knock-out was employed in these studies. The ALK gene targeting vector thus included an FRT site upstream of exon 19 followed by a LacZ cassette and a *lox*P site. Upstream of exon 23 a PGK-TK negative selection gene and a PGK-neo positive selection gene (transcribed in the opposite direction of ALK) were flanked by *lox*P sites. The most upstream *lox*P site was flanked by an FRT site (Fig 1, specific details available upon request). The ALK sequence used for constructing the vector was recovered by screening the Invitrogen RPCI-22M BAC bank, BAC M409A20 (Invitrogen) was sequenced to verify the ALK fragment, and using KpnI/SpeI a region spanning exon 17–24 was subcloned into LITMUS28i (New England Biolabs, Inc,. Berverly MA, USA). The subsequent vector was electroporated into 129/Sv mouse embryonic stem cells prior to selection for neomycin resistance. After transient expression of Flp and negative selection using ganciclovir positive clones (verified via Southern and PCR) were used for injection into C57BL/6N blastocysts, generating chimeric mice and establishing the ALK kinase KO mouse line. ALK genotypes were assessed using genomic DNA isolated from mouse tail biopsies. For Southern blot analysis, genomic DNA was digested with SpeI to generate a 14.8 kbp fragment for the wild type allele and an 8.8 kbp fragment for the targeted allele. Southern blot was conducted according to manufacturer’s protocol (Roche DIG Application Manual), using a digoxigenin (DIG) labeled probe directed against ALK intron 17. For routine genotyping, genomic DNA was amplified by standard PCR using 5´-ATAGTTCGCCAGCGCGCACCGTA-3´ and 5´-TGCAGTCACTGCGAGGTAGACG-3´ for the targeted allele and 5´-CCTGATGATCAGAGCTTC-3´ and 5-GAATCCTCACGATTCTGG-3´ for the wild type allele to yield products of 437 bp and 301 bp, respectively. For the CRE-ALK line, 5´-ATAGTTCGCCAGCGCGCACCGTA-3´ and 5´-CCCTTTCAGAAGCCAGTCCTT-3´ was used for the targeted allele and 5´-CCTAGGAGTGCTAAGACC-3´ and 5´-GAGGTAGACGAATGTCAC-3´ for the wild type allele to yield products of 311 bp and 360 bp respectively. Taq DNA polymerase from New England Biolab (#M0273X) was used. The PCR consisted of an initial incubation step at 94°C for 2 min, followed by 30 cycles at 94°C for 30´, 56°C for 30´ and 72°C for 30´. A last extension step at 72°C for 5 min was performed. Mouse monoclonal antibody #135 used for verification of knock-out animals by immunobloting was generated in our laboratory. The antigen used for immunization of mice was His-tagged extracellular part of human ALK (a kind gift from Marc Vigny). Antibody #135 cross-reacts with mouse ALK. All experiments were done on C57BL/6N background.

### Pubertal onset assessment

After postnatal day 21, preputial separation (PPS) in males was assessed daily in ALK mutant mice as well as in wild type animals (n = 10 from P21 to P40 and n = 6 from P41 to P60). This consisted of attempts to manually retract the prepuce with gentle pressure. PPS is testosterone dependent and thus is an indicator of activation of the reproductive axis in males [24]. Puberty in rodents is dependent on weight [25]; hence, the weights of ALK knock-out mice and control littermates were assessed in prepubertal mice through adulthood.

### Blood collection and testosterone measurements

Mice were briefly anaesthetized with isofluorane and blood samples were collected from the facial vein with Golden rod animal lancet (MEDIpoint) to the BD Microtainer tube with serum separator [26]. Serum samples were frozen and stored at -20°C until analysis. Serum testosterone levels were quantified with the Spectria Testosterone RIA kit (Orion Diagnostica), or with the Testosterone ELISA kit (DRG, USA), according to the manufacturer’s instructions.

### Sperm count

Immediately after sacrifice, the caudal epididymis was removed and placed in a small clean petri dish containing 1ml of PBS pH = 7.4 (n = 4 per age and per genotype). The caudal part was dissected with a sharp sterilized blade into three pieces and squeezed gently with fine forceps to release the sperm, and the suspension was then allowed to settle at 37° C for 30 min [27]. Sperm were counted using a Hemocytometer Chamber. Sperm suspension was placed on both sides of the chamber and the number of sperm counted at 100X magnification[25]. Sperm count was performed in triplicate for each sample (n = 4 at P40 per genotype, n = 6 at P60 for WT animals and n = 4 at P60 for ALK mutant mice) [28].

### Sperm viability

Twenty μL of sperm suspension and 20 μL of eosin solution were mixed and vortexed for 2 sec. After, 30 μL of the suspension were recovered and spread on a glass slide. After drying at room temperature, sperm viability was determined: dead and living spermatozoa had red and white colored heads, respectively. A minimum of 200 spermatozoa was counted in triplicate per sample (n = 4 per age and per genotype).

### Crizotinib treatment

C57BL/6N male mice at the age of 7–9 weeks were obtained from Taconic. They were treated orally for 14 days with crizotinib (20 mg/kg) or vehicle (90% PEG 300, 10% 1-methyl-2-pyrrolidine) (n = 13 per each treated group). Mice were kept in separate cages and briefly anaesthetized with isofluorane for the drug administration. All handling concerning blood collection and treatment was done with an attempt to minimize animals stress as well as cross-contamination with pheromones. No loss of weight or any other visible signs of animals being negatively affected by the treatment were observed during the whole experiment.

### Histology and immunohistochemistry

Collected organs from 10 animals per age and per genotype were fixed in 4% paraformaldehyde overnight. After two washes in phosphate buffer solution (PBS), testes were dehydrated through an ethanol series and embedded in paraffin (Tissue-Tek VIP processor, Sakura). Tissue sections (5–7μm) were mounted on SuperFrost Plus slides (Menzel-Gläser, Thermo Scientific). Slides were deparaffinized in xylene, rehydrated through ethanol series and stained with hematoxylin and eosin. Epitope retrieval was achieved by 3 min boiling in sodium citrate 10 mM pH 6.0 at the microwave. After washing with PBS and blocking for 15 min with 5% bovine serum albumin in PBS with 0.1% Triton X-100, samples were incubated with a primary antibody against GATA4 (Santa Cruz). Primary antibody was detected with the appropriate secondary antibody, coupled to horse radish peroxidase (HRP) (Santa Cruz). HRP activity was detected with the chromogenic substrate ImmPACT DAB (SK-4105, Vector Labs, USA).

### OPT analyses

For optical projection tomography (OPT) [29], brains from male mice at the age of 40 (n = 5 for wild type and n = 6 for ALK mutant mice) and 60 days (n = 7 for wild type and n = 5 for ALK mutant mice) were dissected. Hypothalami with margins of surrounding tissues were cut out, stained for gonadotropin releasing hormone GnRH (PAI-121, Thermo scientific, Pierce antibodies) and processed for OPT imaging essentially as described [30]. OPT scanning and tomographic reconstruction was performed as described [31] implementing algorithms for COM-AR and A-value tuning [32] and contrast limited adaptive histogram equalization [33] using a Bioptonics 3001 scanner (SkyScan). Iso-surface rendering and quantification was performed using Imaris version 7.6.5 (Bitplane).

### Statistical analysis

Statistical analyses were performed with GraphPad Prism 6 software. Mann-Whitney and Wilcoxon tests were applied. *P*-values less than 0.05 were accepted to indicate statistical significance.

## Results

### Generation of the ALK kinase knock-out (KO)

The ALK kinase KO was generated via homologous recombination and subsequent excision of exon 20–23 (described in detail in material and methods). Exons 20–23 correspond to nucleotides 3783–4255 and amino acids 1062–1252 (ALK sequence via NCBI NM_007439). Deletion of these exons disrupts essential structural components of the ALK kinase domain, resulting in a kinase-dead ALK receptor (Fig 1A). Animal genotypes were verified via PCR, southern and western blotting analyses (Fig 1B). Heterozygous intercrosses were performed for the analysis of homozygous mice. Mice of all three genotypes (+/+,-/- and +/-) were obtained in expected Mendelian ratios. Thus, mice homozygous for the ALK kinase deletion are born and are also viable. Homozygous ALK mutant mice of both genders are fertile and were used to establish a colony of homozygous ALK kinase-deficient (ALK kinase KO) mice.

### ALK KO male mice exhibit hypogonadotropic hypogonadism

**Fig 2:**
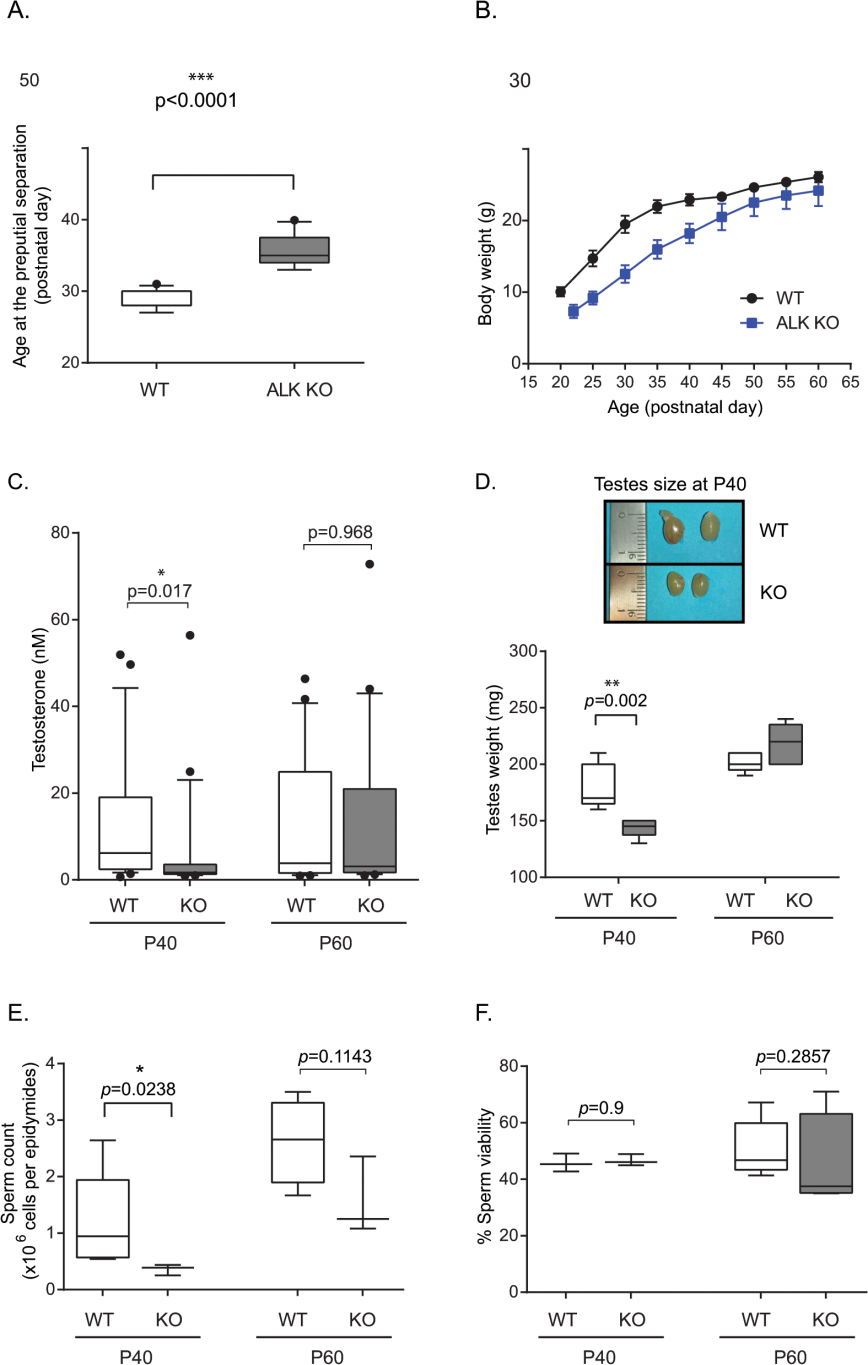
Effect of loss of ALK activity on puberty onset and testosterone levels. (A) The age at preputial separation was assessed in littermate control (n = 11) and ALK mutant (n = 12) male mice. (B) Body weight development in male mice (n = 10 each genotype). (C) ALK kinase KO male mice display significantly lower levels of testosterone at P40 as compared with controls. No significant differences between ALK kinase KO and wild type were observed at P60. Box plots indicate the median (black line), 25th and 75th percentiles (borders of boxes), 10th and 90th percentiles (whiskers) and the outliers (dots) from 20–25 mice per group. *P<0.05 indicates significant difference. (D) Testicular weight (n = 5 per postnatal day and genotype), as well as representative testicular size in wild type mice compared with ALK KO mice is shown. (E) Number of cauda epididymidis-retrieved spermatozoa per epididymis (n = 4 per genotype at P40, n = 6 at P60 for WT animals and n = 4 at P60 for ALK mutant mice). (F) Viability percentage of cauda epididymidis-retrieved spermatozoa (n = 4 per genotype and per age).

The kinase-domain KO ALK mice described here do not display any gross phenotypes, in agreement with ALK mutant mouse models reported earlier [12, 13]. However, during breeding of these mice we observed that ALK kinase knock-out mice initiated breeding at later time points as compared with wild type mice. In order to investigate why the ALK kinase KO mice breeding patterns are delayed we assessed multiple parameters. Pubertal onset in ALK knock-out mice was assessed in males by preputial separation (PPS). PPS was delayed by approximately 5–7 days in ALK knock-out mice (postnatal day 35 ± 0.67) compared with that in wild type mice (postnatal day 28 ± 0.82, *P* = 0.0001; Fig 2A). Since puberty in rodents is dependent upon weight [34], the peripubertal weight of ALK knock-out and control littermates was assessed. The weight of ALK knock-out and control mice was significantly different at P40 (*P* = 0.001) but not at P60 (*P* = 0.08; Fig 2B). We then measured levels of serum testosterone at both 40 and 60 days after birth (P40, P60) in blood samples collected from wild type (n = 26 per age) and homozygote ALK mutant male mice (n = 24 per age).

In contrast to wild type mice at P40, ALK kinase KO male mice showed significantly lower levels of serum testosterone (*p*-value = 0.017). At P60 we were unable to detect significant differences between serum samples from wild type and ALK kinase KO males (Fig 2C). This decrease in serum testosterone levels in ALK mutant males at P40 supports our observation that ALK mutant mice exhibit hypogonadotropic hypogonadism as compared to their wild type littermates. Testicular weights and sizes were also determined at P40 and P60, as testicular weight increases at the time of puberty. At P40, the testicular weights of wild type mice were significantly greater than those of ALK kinase KO mice (180 mg vs 143 mg, *P* = 0.002; Fig 2D). This is associated with bigger testes size in wild type mice compared with ALK mutant mice. This difference disappears by P60, when the testicular weights of ALK mutant mice were no longer significantly different from those of wild type mice. Sperm counts, following sperm retrieval from the cauda epididymides, show a significant decline (*p*-value = 0.0238; Fig 2E) between WT and ALK kinase KO mice at P40 (1.23x10^6^ versus 0.36 x10^6^ sperm/epididymides) but not at P60 (*p*-value = 0.1143; 2.33x10^6^ versus 1.56x10^6^ sperm/epididymides). However, sperm analyses parameters revealed no significant changes in sperm viability (Fig 2F) in ALK kinase KO mice compared to their corresponding controls at either P40 (*p*-value = 0.9) or P60 (*p*-value = 0.2857).

**Fig 3:**
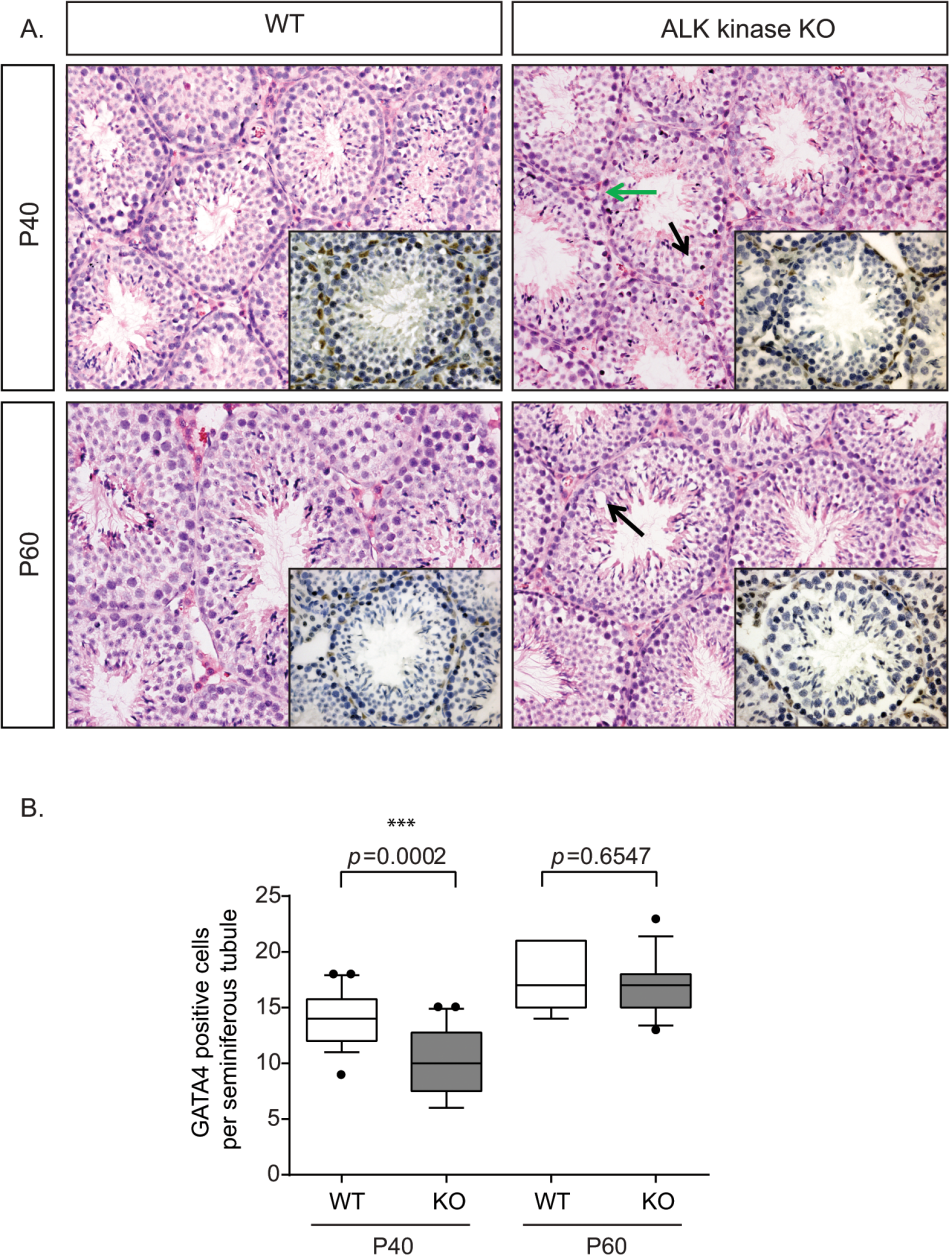
Abnormalities in seminiferous tubules from ALK kinase KO testes. (A) Histological morphology of testes stained with standard H & E. Note the irregular arrangement of germ cells at the periphery (green arrow) of the seminiferous tubule and the increased presence of vacuoles (black arrow) in ALK kinase KO mice. Fewer GATA4-expressing cells within the seminiferous tubules are observed at P40 in ALK kinase KO mice as compared with wild type controls. This decrease is less obvious at P60 (insets). (B) Quantification of age-dependent loss of GATA4 immunoreactivity in the Sertoli cells within the seminiferous tubules of wild type and ALK kinase KO mice. N = 10 mice per age and per genotype.

To verify if decreased testosterone levels are associated with any abnormal testicular histoarchitecture, we performed standard hematoxylin and eosin staining of paraffin sections of testes collected from ALK KO mice and wild type siblings at P40 and P60. As the number of Sertoli cells per seminiferous tubule depends on the stage of the epithelial cycle and the shape of the section, we ensured that the investigated tubule cross-sections were absolutely round and were not close to the turn of the tubule to allow proper quantification. Seminiferous tubules of wild type mice consist of well-organized layers of germ cells and Sertoli cells in the peripheral layer. However, germ cells with irregularly arranged nuclei (Fig 3, green arrow) and with fewer Sertoli cells as visualized by GATA4 immunostaining within the seminiferous tubules at P40 (Fig 3A, insets) were observed in the ALK KO mouse testes. Reduced nuclear hyperchromasia, especially prominent at the outer cell layers, coupled with disturbed cell polarity and cell shrinkage were other striking features. Intracytoplasmic vacuoles coupled with apoptotic bodies were major cytological abnormalities. These vacuoles appeared abundantly at P40 and occasionally at P60 within the tubules of the ALK KO mice testis and were rarely observed in wild type testes at P40 (Fig 3A, black arrows). The presence of vacuoles and apoptotic bodies may be indicative of degenerative changes and/or disturbed cell maturation. We further performed a GATA4-positive cell count and showed that the number of GATA4-expressing Sertoli cells within the seminiferous tubules in ALK KO mice was significantly lower than that in the wild type animals examined at P40 (*P* = 0.0002) but not at P60 (*P* = 0.6547; Fig 3B). Taken together, these data suggest that ALK KO mice are delayed in their sexual maturation, due in part to decreased testosterone levels and mild testicular disorganization.

### Inhibition of ALK reversibly decreases testosterone level in male mice

**Fig 4:**
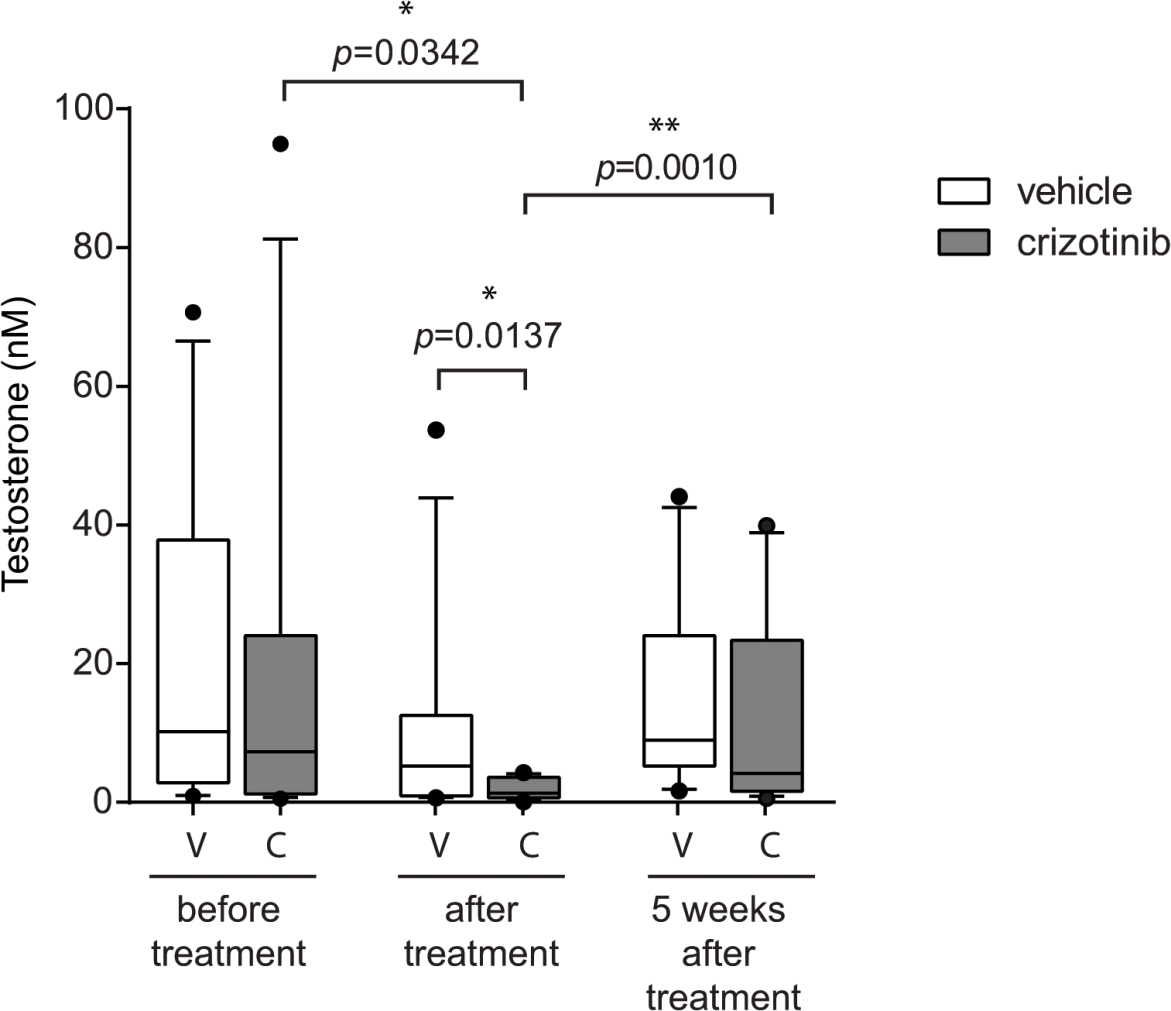
Crizotinib treatment in mice leads to reduction of serum testosterone levels. Adult male mice were treated with crizotinib daily (at 20 mg/kg). Blood samples were collected prior to crizotinib treatment, one day after the last treatment and five weeks after completion of treatment and analyzed for testosterone. Box plots indicate the median (black line), 25th and 75th percentiles (borders of boxes), 10th and 90th percentiles (whiskers) and the outliers (dots) from 13 mice per group. *P<0.05 indicates significant difference.

These observations are interesting given recent reports of the effects of crizotinib on male NSCLC patients in which a rapid suppression of testosterone levels was observed in men under crizotinib treatment [17]. Our findings in ALK KO mice prompted us to examine whether a similar phenomenon could be observed in wild type mice in response to crizotinib. To test this hypothesis, 26 adult male mice were randomized in two groups: treated and control. Mice aged from 7 to 9 weeks old were treated daily with 20 mg/kg crizotinib or vehicle for two weeks. Three independent blood samples were collected from each group: one prior to treatment, a second directly after the end of treatment and a final sample 5 weeks post-treatment and analyzed for serum testosterone levels (Fig 4). The results show significantly decreased testosterone levels in mice after crizotinib treatment. However, this decrease was reversible, since 5 weeks after crizotinib cessation testosterone levels were once again comparable to normal control levels. No significant changes in testosterone levels were observed in control group mice. The observed changes in testosterone levels display a rapid onset, returning to normal upon termination of treatment, confirming a direct causal relationship with crizotinib administration. Thus, treatment of mice with crizotinib results in lower levels of serum testosterone, suggesting that crizotinib treatment abrogates testosterone production in male mice.

### Decreased levels of gonadotropin releasing hormone (GnRH) in ALK KO mice

**Fig 5:**
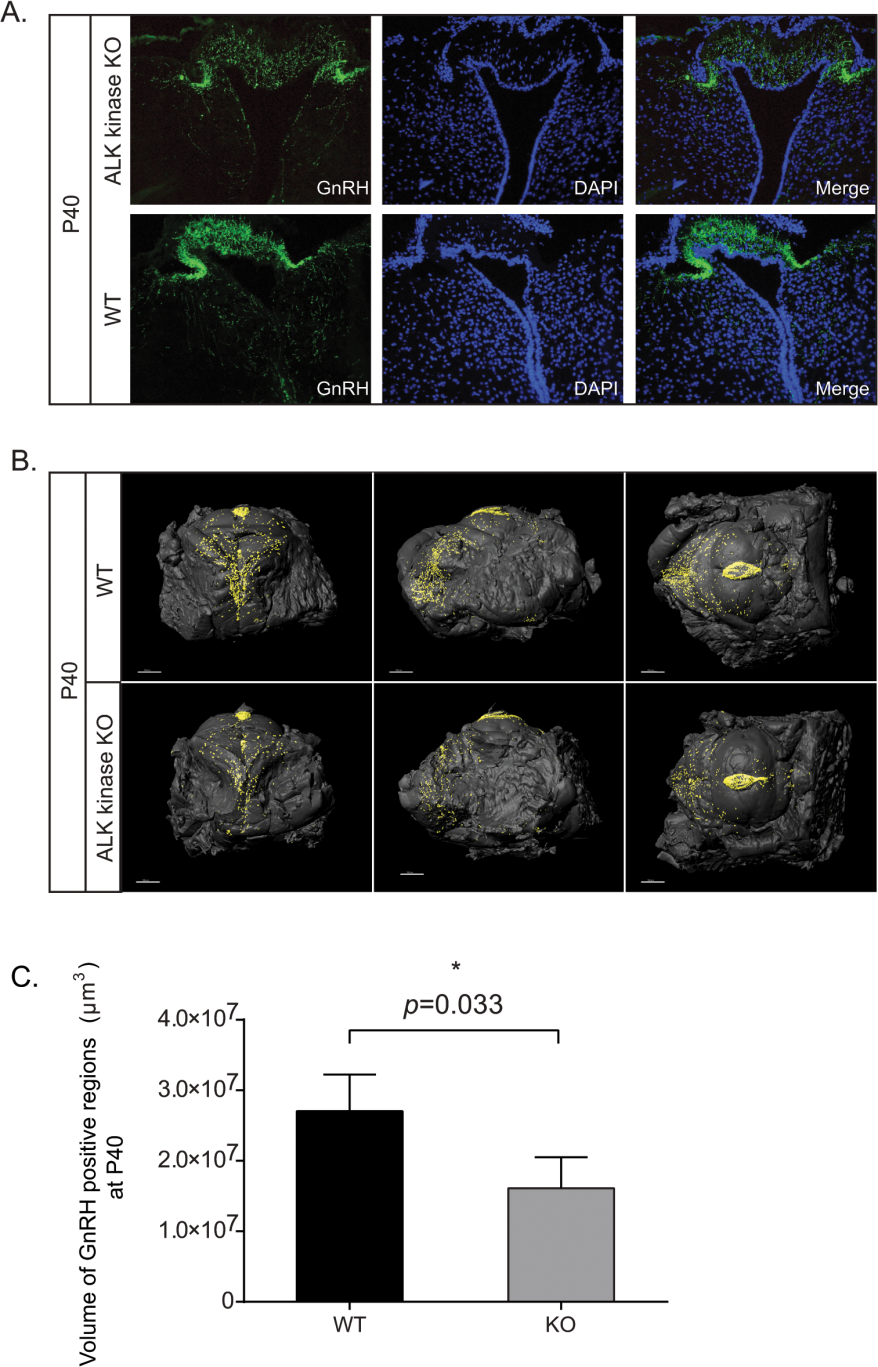
ALK knockout mice display reduced GnRH expression. (A) Confocal analysis of GnRH expression in P40 ALK kinase KO. GnRH (green), DAPI (blue). (B) Expression of hypothalamic GnRH at P40 in ALK kinase knockout males. Reconstituted OPT images of representative hypothalami are shown in anterior, lateral and ventral views. GnRH positive regions are presented as (iso-surface rendered) black dots on the iso-surfaces of dissected hypothalami. (C) Quantitation of GnRH positive regions from P40 hypothalami. Values represent the average volumes of GnRH stained regions in hypothalami, 3–4 mice per group. *P<0.05 indicates significant difference.

It has been postulated that the crizotinib induced reduction of testosterone levels in human males may be due to hypothalamic or pituitary effects in response to treatment with crizotinib [17]. In order to test this, expression of GnRH positive neurons in ALK KO was examined. Levels of GnRH in ALK KO hypothalamic slices were lower than those observed in control mice (Fig 5A). To examine this more carefully, hypothalami collected from wild type and ALK KO mice at P40 and P60 was evaluated by optical projection tomography (OPT), allowing direct quantification and three dimensional–spatial assessments of GnRH expressing cells. A clear reduction in the volume of GnRH positive cells was observed in ALK KO mice at P40 when compared with wild type mice; such reduction in GnRH expression was not observed at P60 (Fig 5B, quantified in 5C; S1 Fig, quantified in S1). Taken together, examination of hypothalamic GnRH levels by both convention confocal microscopy and OPT analysis indicates a role for ALK in the modulation of the hypothalamic-pituitary-gonadal axis affecting hypogonadotropic hypogonadism.

## Discussion

Proper sexual maturation is required to support essential reproductive processes such as the estrous cycle and ovulation in females and the completion of spermatogenesis in males [35]. A number of key players within the hypothalamic-pituitary-gonadal axis are involved in this complex process, such as hypothalamic GnRH, pituitary gonadotropins and the gonadal sex steroids e.g. testosterone (reviewed in [36]). In this article we report the characterization of ALK kinase KO mice, which are generally viable and fertile, in agreement with previous observations [12, 13, 37]. Reported phenotypes in independent ALK KO mouse models include regulation of dopamine signaling in the frontal cortex [12], reduced neurogenesis and enhanced performance in spatial memory in ALK mutant mice [13] and a role for ALK in behavioral responses to ethanol and cocaine [37]. In our examination of ALK kinase KO mice we chose to focus on an early observation of delayed breeding in the ALK kinase KO colonies. An additional ALK KO mouse model, in which exon 1 of *ALK* was deleted, was also examined and found to be viable but also displayed delayed breeding. This observation was strengthened by the findings that ALK kinase KO male mice exhibit significantly lower serum testosterone levels at P40 when compared with control litter mates. Interestingly, these decreased testosterone levels are accompanied by mild histoarchitectural changes in the testes from these mice, including appearance of vacuolated nuclei in seminiferous tubules, decreased numbers of Sertoli cells and irregularly distributed germ cells. These findings were further strengthened by the finding that ALK kinase KO mice have decreased number of GATA4-expressing cells within the seminiferous tubules. GATA4 is expressed in Sertoli and Leydig cells and plays an important role in-testes development and also in Sertoli cell function in adult mice [38]. Indeed, adult mice lacking GATA4 in the testes also exhibit vacuoles in the seminiferous tubules [38]. Examination of sperm in ALK kinase KO mice reveals a significant decrease in sperm count, although viability was not affected. Given the reduced testosterone levels and the decreased sperm count in ALK kinase KO mice, it would appear that loss of ALK activity may lead to suboptimal testes function. While we noted clear differences between ALK kinase KO and wild type mice at P40, it would be of interest to examine this further at earlier time points in future studies as the assessment of puberty onset by preputial separation indicates that the puberty, while delayed in ALK kinase KO mice, occurs between P35-40.

To further examine this we employed ALK kinase inhibitors, in this case the ATP analogue crizotinib [14], to modulate ALK activity in wild type adult mice. As would be predicted from our findings in the ALK kinase KO animals, treatment with crizotinib resulted in reduction in serum testosterone levels. Some depression of testosterone levels was also observed in control mice after feeding with vehicle, which may be due to use of anesthetic during administration or animal handling[39]. Furthermore, these effects were reversible, with serum testosterone returning to normal levels upon cessation of crizotinib treatment. These results are interesting in light of a recent report of rapid-onset hypogonadism secondary to crizotinib treatment in male patients treated for ALK positive non-small cell lung cancer [17]. Thus, while crizotinib is not absolutely specific for ALK among the tyrosine kinase family and targets other than ALK must be considered in these patients, our findings in ALK KO mice suggest that ALK inhibition in patients may result in modulation of testosterone levels.

These findings also have implications for patients being treated with other ALK inhibitors, several of which are now in clinical use [40], including the recently FDA approved ceritinib (LDK378) [41, 42]. A young male ALK positive NSCLC patient treated with crizotinib, and subsequently with ceritinib, is of particular interest. This patient displayed reduced plasma testosterone levels which were treated effectively with testosterone supplement. He later became crizotinib resistant and continued ALK inhibitor treatment, in this case with ceritinib, but continues to require testosterone supplement (Helland, Å., personal communication). Since ALK positive lung cancer patients are generally younger and fitter than the majority of lung cancer patients, low levels of testosterone can induce fatigue and reduced libido and impact on quality of life. For patients living with cancer as a chronic disease, quality of life is extremely important, thus clinicians following these patients should be aware of this potential effect and more knowledge is required concerning ALK inhibition in this patient group. Other relevant patient groups are young neuroblastoma patients, as well as other pediatric cancer patients with ALK driven cancers, that may take ALK inhibitors on a long-term basis [43]. Furthermore, information on testosterone levels in patients taking ALK inhibitors targeting other alterations in cancer, for example MET and ROS, should also be gathered.

While ALK is expressed in testes [11], the decrease of FSH and LH in patients treated with crizotinib suggest that decrease of testosterone may be centrally mediated [17]. LH and FSH are produced by the anterior pituitary in response to GnRH secreted by neurons in the hypothalamus into the portal blood system [44]. Our initial examination of GnRH levels in the hypothalamus of ALK kinase KO mice identified decreased levels of GnRH in ALK mutant mice. More accurate Optical Projection Tomography analysis allowed quantification and confirmed significantly lower GnRH expression levels in the hypothalamus of ALK kinase KO males at P40 as compared with control littermates. These findings support the hypothesis that downregulation of testosterone level either by chemical or genetic depletion of ALK is at least in part initiated upstream in the hypothalamic pituitary gonadal axis. Thus our mouse ALK knockout model displays hypogonadotropic hypogonadism. A number of additionally interesting regulators of hypogonadotropic hypogonadism exist, including e.g. the kisspeptins [45]. Kisspeptins bind to the GPR54 receptor on the GnRH-neurons regulating the pulsing of GnRH secretion [46]. While we have not investigated Kisspeptin in this study, it will be interesting to study them further in the context of ALK function.

Here we show that mice lacking ALK function exhibit a delay in testosterone production with a decreased sperm count. Further, wild type mice treated with the ALK inhibitor crizotinib display robust reduction in testosterone levels in response to drug administration. These results, together with the decreased expression of GnRH in the hypothalamus of ALK KO males indicate a novel role for the ALK receptor tyrosine kinase in the regulation of hypogonadotropic hypogonadism, functioning in the hypothalamic pituitary gonadal axis.

## Supporting Information

S1 Fig OPT analysis of hypothalamic GnRH at P60. Expression of hypothalamic GnRH at P60 in ALK kinase KO analyzed by OPT. GnRH positive regions are presented as black dots on the iso-surfaces of dissected hypothalami. Quantitation of GnRH positive regions from P60 hypothalami. Values represent the average volumes of GnRH stained regions in hypothalami. *P<0.05 indicates significant difference.
(PDF)
