# Supplementary figures and images for: Targeted Disruption of ALK Reveals a Potential Role in Hypogonadotropic Hypogonadism
Source: PLoS One. 2015 May 8;10(5):e0123542. doi: 10.1371/journal.pone.0123542 (PMC4425494; doi:10.1371/journal.pone.0123542)

A

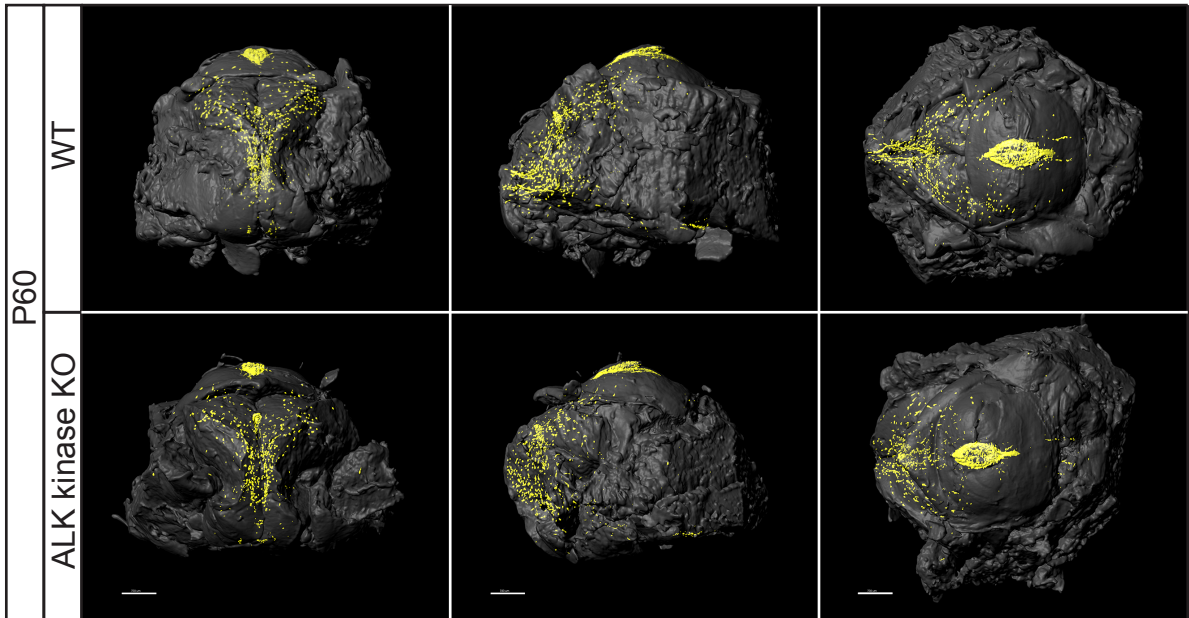

B

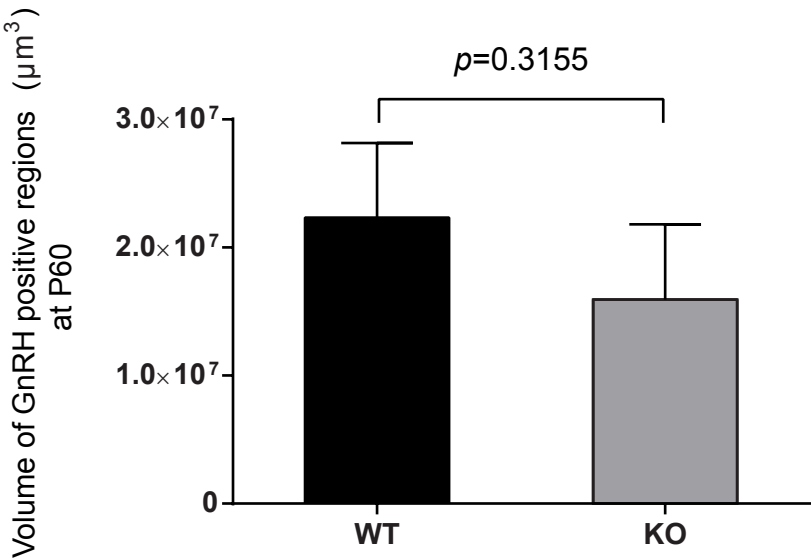

Supplement: S1 Fig — Expression of hypothalamic GnRH at P60 in ALK kinase KO analyzed by OPT. GnRH positive regions are presented as black dots on the iso-surfaces of dissected hypothalami. Quantitation of GnRH positive regions from P60 hypothalami. Values represent the average volumes of GnRH stained regions in hypothalami. *P<0.05 indicates significant difference. (PDF) [file pone.0123542.s001.pdf]
